# Sexual Self-Concept Differentiation: An Exploratory Analysis of Online and Offline Self-Perspectives

**DOI:** 10.3390/ijerph19126979

**Published:** 2022-06-07

**Authors:** Alexandru Mateizer, Andra Cătălina Roșca, Eugen Avram

**Affiliations:** 1 Department of Psychology, Faculty of Psychology and Educational Sciences, University of Bucharest, 050663 Bucharest, Romania; eugen.avram@fpse.unibuc.ro; 2 Department of Sociology, Faculty of Political Sciences, National University of Political Studies and Public Administration, 012104 Bucharest, Romania; catalina.rosca@politice.ro

**Keywords:** sexual self, self-concept differentiation, online sexual activities, sexual identity

## Abstract

Self-concept differentiation (SCD) has been of interest to researchers, mainly as a structural concept indicative of social specialization or self-concept fragmentation. Nevertheless, this aspect of self-representation has not been studied in regard to sexuality and the extent to which the sexual self may vary across different roles or situations. With the emergence of the Internet, people found new opportunities to explore and express aspects of their sexuality in multiple online scenes, thus increasing the complexity of human sexual experience and expanding the reach of sexual identity. The aim of this study is to investigate SCD in relation to the sexual self-concept, as experienced in the online and offline environments, and its effects on sexual identity, sexual satisfaction and online sexual behaviors. Data analysis pointed towards a fragmented self-view with high degrees of differentiation between the online and offline sexual self-instances being linked to a weaker sense of sexual identity, less sexual satisfaction in real life and less partnered online interactions. However, there were some indications that these relationships were influenced by how people perceive themselves sexually in one instance compared to the other. The results obtained in this study encourage further research on SCD as an important factor in understanding the real-world consequences of online sexual expression.

## 1. Introduction

Sexuality, although an essential component of the self, can be difficult to express and explore, even in the context of an intimate relationship. For many, sexual desires and fantasies are often accompanied by embarrassment or muted by fears of rejection and unpleasant reactions. It could be argued that lacking exploration and acceptance of such aspects of one’s self may lead to variants of potential sexual self-instances that remain isolated in fantasized experience. Such pockets may lead the individual to acquire a sense of partiality or even fragmentation in regard to their sexual self, thus limiting the possibility of a more cohesive self-experience. However, with the increasing technological complexity and expansion of the online environment came new opportunities for self-expression, but also new challenges of adapting to new roles and experiences. In this situation, the multiplicity of selves that emerge according to context are reflective of the individuals’ various involvements and are subjected to efforts of synthesis. To date, the research on sexuality and the Internet has mainly been focused on associated risks and various harmful outcomes related to gender attitudes [1,2], uncommitted sex [3,4], psychosocial functioning, addiction and compulsivity [5,6,7], with very few studies investigating positive implications for people’s sex lives [8,9]. In order to further pursue the interaction between sexuality and the online environment, this study focuses on the continuity between online and offline sexuality and the roles of online sexual activities and sexual self-concept differentiation across these two dimensions.

In contrast to the risk-focused literature and established negative outcomes of online sexuality, a series of studies on the aspects of online sexual self and social identity [10,11,12] have found many beneficial aspects for those involved in online sexual interactions, in particular, for those who found it easier to express their sexuality online rather than offline. The virtual world seems to offer a safer environment for exploring one’s sexuality and an opportunity to identify with like-minded people [13], as well as being a place to experiment with flirtation, sex roles and different personas [14]. The positive outcomes included more self-acceptance of sexuality, increased sexual self-confidence in real life and identity transformation. Additionally, moderate engagement in online sexual activities (OSAs), such as pornography use, were found to have positive influences on sexual self-esteem for males [15] and yielded certain benefits for both males and females, especially within intimate relationships, including increases in frequency of intercourse and better sexual communication [16]. However, given the known risks and negative impacts on real life that are associated with online sexuality, addressing the continuity between these two sexual domains becomes a complex multi-determined process. Using the self-concept differentiation (SCD) model, developed by Donahue and collaborators [17], this study aims to further expand the knowledge about the Internet and sexuality by exploring how people observe themselves sexually in the online environment, and how this particular aspect of sexuality relates to a more general sense of sexual self. The notion that the self is a multifaceted cognitive structure [18,19,20], containing multiple self-aspects, has been subject to extensive empirical research that is mainly focused on determining the relationships between various indices of maladjustment, such as emotional distress [21,22] or identity [10,23,24], and a divided self-concept, which lacks integration (i.e., self-concept fragmentation). However, according to some theories of the self, the distinction among self-aspects is thought to have both adaptive and stress-buffering qualities [25,26], reflecting self-concept complexity and specialization. One of the most widely used models in this regard is SCD (i.e., lack of interrelatedness of roles), which expresses people’s tendency to view themselves as having different personality characteristics across different social roles [23]. The SCD index proposed in this model reflects the differences among the rated characteristics for different roles and can be obtained by measuring the unshared variance between roles or absolute differences among the roles. Using the SCD framework, we seek to investigate the possible role of this phenomenon in relation to the interaction between online and offline sexuality.

Given the opportunity for people to explore and express aspects of their sexuality in multiple online scenarios, such as various interactive platforms or websites, the Internet has become a place where one can experiment with sexual identity. In the SCD framework, this process could be understood as movement towards a less fragmentated sexual self and consequently towards more consistency in how one experiences sexuality online and in real life, by maintaining a core sense of sexual self. However, the SCD model is currently not employed in sexuality research and, therefore, the possible effects related to this concept and its validity remain unknown.

In conclusion, this study aims to expand the knowledge on contemporary sexual behaviors and to narrow the gaps in the literature by addressing SCD in the context of online sexuality and taking into account online sexual activities (OSAs; i.e., Internet pornography and sex chat), sexual identity and sexual satisfaction. By employing the SCD framework, we attempt to better understand how people experience their sexual selves in two different settings, online and real life, and to investigate the relationships between these self-instances and the measured outcomes. Additionally, we aim to better understand the degree to which the two methods of computing SCD (non-shared variance and absolute differences) overlap and whether or not they reflect the same phenomenon.

## 2. Materials and Methods

### 2.1. Participants and Procedure

The present research was conducted based on a sample of 106 respondents consisting of 59 females and 47 males, with a mean age of 24.44 years (SD = 6.35). Recruitment for this study was conducted through social media platforms, such as Facebook, where invitations were offered for participation. The questionnaire application process involved the completion of an online form comprised of several sections: information regarding the confidentiality of personal data, informed consent and the measures used in the study.

SCD. At the beginning of the questionnaire, respondents were given a definition of the sexual self-concept and were asked to indicate how they felt about their sexual selves as experienced in the online environment. A total of 18 pairs of bipolar adjectives were presented to them for this purpose, rated on a 7-point Likert scale (e.g., relaxed = 1 2 3 4 5 6 7 = tense). To minimize the carry-over effects, the procedure was repeated at the end of the questionnaire, this time by referring to their real-life experiences. The computation of SCD was performed based on two methodological approaches, described in the Data Analysis section.

### 2.2. Measures

Online sexual activities (OSAs). A series of items were developed in a previous study [27] in order to assess the frequency of engagement in online sexual activities in the previous 6 months. A total of 6 items (α = 0.778) was used, measuring 2 types of online sexual activities dating back 6 months: solitary OSAs (2 items; e.g., “I watched sexually explicit material on the Internet”) and partnered OSAs (3 items; e.g., “I have exchanged intimate photos online with another person”). Each item was scored on a 6-point Likert scale (1 = not at all; 6 = almost daily), and the final scores were obtained by summing the corresponding items.

Sexual Self-Concept (SS). The sexual self-concept was measured, for both online and offline instances, using The Meaning of My Sexual Self scale [28]. This measure included 18 items (α = 0.945) consisting of pairs of bipolar adjectives rated on a 7-point Likert scale. The items reflected three dimensions: evaluation (e.g., “valuable–worthless”), potency (e.g., “strong–weak”) and activity (e.g., “involved–uninvolved”). The final score was obtained by summing all the items.

Sexual Identity. Sexual identity was evaluated using The Measure of Sexual Identity Exploration and Commitment [29]. The scale consisted of 22 items (α = 0.820) grouped into 4 subscales: exploration (8 items; e.g., “I am actively trying to learn more about my own sexual needs”), commitment (6 items; e.g., “I know what my preferences are for expressing myself sexually”), sexual-orientation uncertainty (3 items; e.g., “My sexual orientation is clear to me”) and synthesis (5 items; e.g., “My sexual orientation is compatible with all of the other aspects of my sexuality”). Each item was scored on a 6-point Likert scale (1 = very uncharacteristic of me; 6 = very characteristic of me). The final scores were obtained by summing the corresponding items.

Sexual satisfaction. Satisfaction with real-life sexual intercourse was measured with The New Sexual Satisfaction Scale-Short Form (NSS-S) [30]. This scale consisted of 12 items (α = 0.920) that measured 2 aspects of sexual satisfaction: ego-focused (6 items; e.g., “The way I react sexually to my partner”) and partner and activity-focused (6 items; e.g., “My partners’ letting go during sex”). Each item was scored on a 5-point Likert scale (1 = not at all satisfied; 5 = very satisfied). The total score was obtained by summing the corresponding items.

### 2.3. Data Analysis

Statistical analyses were performed using IBM SPSS statistics (v. 26.0.0.0) (Armonk, NY, USA) [31]. First, SCD was computed based on two methodological approaches. The first method was proposed by Donahue and collaborators [17] and it measured an index of absolute differences among the online and offline sexual-self scores. For each subject, 18 standard deviations were computed across the two scenarios, one for each sexual-self attribute. The end result was obtained by averaging the 18 standard deviations and was used as a measure of distance between the online and offline sexual-self instances. The second method was proposed by Block [32] and it measured the amount of non-shared variance between the different scenarios. For each subject, we intercorrelated the online and offline sexual-self instances across the 18 attributes and extracted the shared variance. The remaining variance was used as a measure of the degree of inconsistency between the online and the offline sexual-self scenarios. Next, in order to evaluate the effects of SCD on the relationships between variables, a series of regression analyses were performed, with SCD parameters as moderators. Finally, we calculated a binary categorical variable labeled “Location of Sexual-Self” (LSS) that grouped the respondents as “Online”, if they scored higher on the online SS relative to the offline SS (n = 32), and “Offline” if they scored higher on the offline SS relative to the online SS (n = 63) (subjects with equal scores on both evaluations of the sexual self-concept (n = 11) were removed from the analysis as they offered no pertinent information in this regard). In order to evaluate the effects of this grouping, an analysis of variance (ANOVA with post hoc Tukey and Games-Howell tests) was performed, followed by a series of regression analyses using the grouping variable LSS as moderator.

## 3. Results

### 3.1. Descriptive Statistics

The descriptive statistics, including the means, standard deviations and correlations of the study variables, can be found in Table 1. Contrary to the previous findings [17,23,33,34], the sexual SCD parameters were not associated with any of the identity related outcomes nor to sexual satisfaction. However, the sexual SCD inconsistency parameter was negatively related to partnered OSAs (r = −0.239, *p* < 0.05), indicating that less overlap between the two sexual-self instances, online and offline, was associated with less frequent engagement in cybersex. Additionally, the association between the two SCD parameters was lower than that obtained by Donahue and collaborators [10] (r = 0.593 < 0.8). Regarding SS, we found that greater sexual SCD was associated with less positive online SS. Compared to SCD inconsistency, the results show a stronger negative relationship between SCD distance and the online SS (r = −0.677, *p* < 0.001), but no significant association with offline SS was found. Additionally, increases in SS were associated with more sexual satisfaction and a stronger sense of sexual identity. Finally, solitary OSAs were positively associated with sexual exploration (r = 0.202, *p* < 0.05), sexual-orientation uncertainty (r = 0.235, *p* < 0.05) and negatively associated with offline SS (r = −0.197, *p* < 0.05), while partnered OSAs were related only to sexual exploration (r = 0.232, *p* < 0.05).

### 3.2. SCD Moderation Analysis

**Figure 1:**
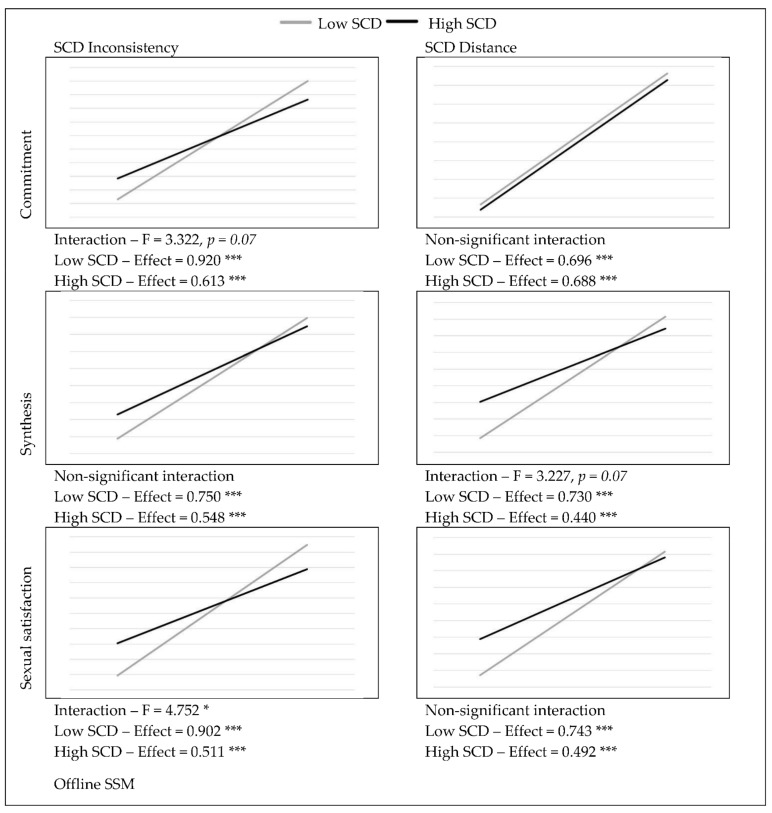
Effects of offline SS on Sexual identity and Sexual satisfaction (standardized means). * *p* < 0.05, *** *p* < 0.001.

The possible moderating role of SCD was investigated in relation to offline SS. In order to test for effects, the PROCESS macro (v4.0) (New York, NY, USA) for SPSS [35] was used, with 5000 bootstrap samples and standardized values. The analysis revealed that that SCD had a buffering effect on the relationships between offline SS, sexual identity and sexual satisfaction (see Figure 1). For higher levels of SCD, the effects of offline SS on commitment to a sexual identity and sexual-identity synthesis decreased. However, the results indicate that the SCD parameters impacted different relationships. For instance, SCD inconsistency buffered the effect on commitment to a sexual identity (F = 3.322, *p* = 0.07), while SCD distance buffered the effect on sexual-identity synthesis (F = 3.227, *p* = 0.07) suggesting that different aspects of SCD might influence different outcomes.

### 3.3. Analysis of Variance

Data analysis showed that some respondents reported feeling more confident and comfortable sexually in the online environment as opposed to real life. This prompted the calculation of a binary categorical variable, labeled “Location of Sexual-Self” (LSS), which grouped the respondents as “Online” if they scored higher on the online SS relative to the offline SS (n = 32), and “Offline” if they scored higher on the offline SS relative to the online SS (n = 63) (subjects with equal scores on both evaluations of the sexual self-concept (n = 11) were removed from the analysis as they offered no pertinent information in this regard).

Between-group analysis (see Table 2) revealed that the subjects whose stronger sense of sexual self was located in the online environment (LSS online) were likely to feel less committed to a sexual identity (F = 3.829, *p* = 0.05), less satisfied with their personal experience and sensations during sex (F = 3.808, *p* = 0.05) and to feel less positive about themselves sexually in the offline environment (F = 20.983, *p* < 0.001), as opposed to subjects in the LSS offline group. Although other comparisons did not reach statistical significance, the results show that individuals whose stronger sense of sexual self was located in offline (LSS Offline) reported higher levels of overall sexual satisfaction, less sexual orientation uncertainty and a higher frequency of engagement in partnered OSAs. The sexual SCD parameters did not differ significantly, both groups showing a similar degree of inconsistency and distance between the two sexual-self instances, with the LSS Offline group reporting slightly higher mean scores, therefore more separation between the two sexual-self instances.

### 3.4. LSS Moderation Analysis

**Figure 2:**
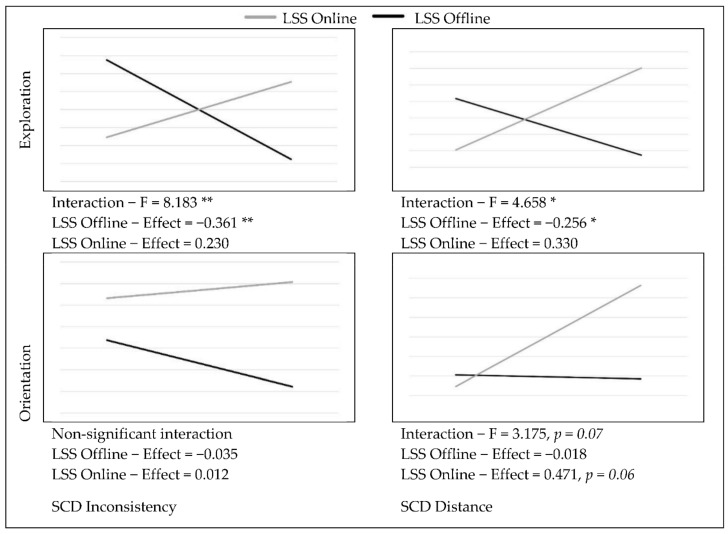
Effects of SCD on Sexual Identity (standardized means). * *p* < 0.05, ** *p* < 0.01.

**Figure 3:**
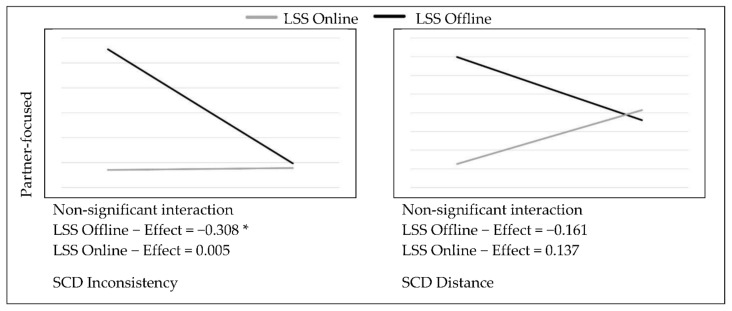
Effects of SCD on sexual satisfaction (standardized means). * *p* < 0.05.

**Figure 4:**
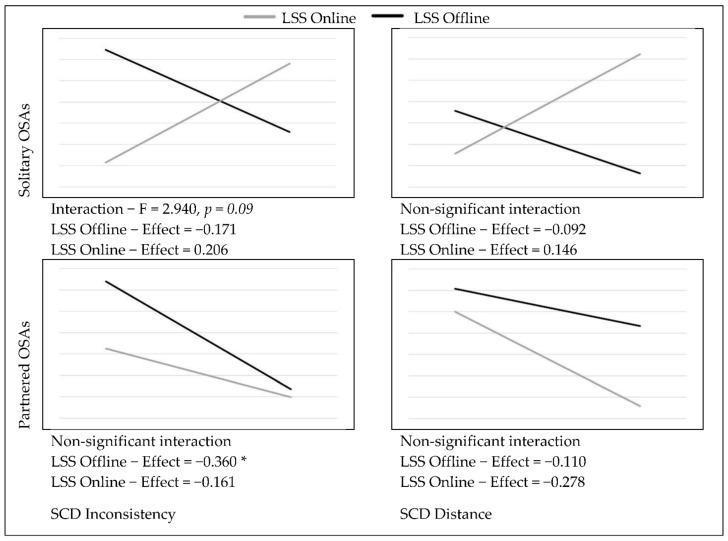
Effects of SCD on online sexual activities (standardized means). * *p* < 0.05.

In order to test the possible moderation effect of the LSS group variable on the relationships between SCD and the measured variables, the PROCESS macro (v4.0) (New York, NY, USA) for SPSS [35] was used, with 5000 bootstrap samples and standardized values. The SCD inconsistency-by-LSS interaction was significant in predicting the interest towards sexual exploration (F = 8.183, *p* < 0.01), as well as marginally significant in predicting solitary OSAs (F = 2.94, *p* = 0.09). On the other hand, the SCD distance-by-LSS interaction was significant in predicting the interest towards sexual exploration (F = 4.724, *p* < 0.05) and sexual-orientation uncertainty (F = 3.175, *p* = 0.07). Simple slope analysis revealed that, among individuals in the LSS offline group, the higher the sexual SCD scores, the lower the interest in sexual exploration (see Figure 2). For the LSS online group, significance was marginally reached only in relation to SCD distance, where higher scores predicted increases in sexual-orientation uncertainty (see Figure 2). Additionally, a non-trivial positive effect of SCD on the interest in sexual exploration was observed for this group, but it did not reach statistical significance (β = 0.330). Additionally, for individuals in the LSS offline group, higher SCD inconsistency scores predicted less frequent engagement in OSAs. The effect of SCD distance on partnered OSAs was smaller and non-significant, suggesting that the engagement in sexual activities with other people online is not only linked to having a strong sense of online sexual self, but rather to a more integrated sexual self-concept that allows for sexual expression. For individuals in the LSS online group, the engagement in solitary OSAs seemed to follow an opposite trend, although the effect was not statistically significant (see Figure 3). In regard to SCD predicting sexual satisfaction, no significant differences between groups were found. However, for individuals in the LSS offline group, the higher the SCD inconsistency scores, the lower the individuals’ satisfaction with how their partners react to them sexually (see Figure 4).

## 4. Discussion

Although previous studies have investigated the effects of self-concept differentiation on various sexual outcomes [36,37,38,39], the possibility of variety in sexual selves has not been researched within the self-fragmentation/specialization framework. This research adds to the existing literature by addressing SCD and its associations with sexual identity, sexual satisfaction and online sexual activities in the context of online sexuality. The main research interest when investigating both self-differentiation and online sexuality has been focused on establishing positive or negative links with various health- and well-being-related outcomes. However, the primary goal of the present study was not to replicate such relationships, but rather to determine if meaningful conclusions can be drawn in relation to applying SCD on the sexual self-concept. More specifically, we looked to see if particular sexual-self views impacted the effect of self-concept differentiation on various sexuality-related outcomes and whether the two methods of determining self-concept differentiation (non-shared variance and absolute differences) expressed two separate dimensions that might be linked to different outcomes. To achieve this, self-concept differentiation was operationalized in terms of relatedness and distance perceived between the online and offline sexual-self instances, and a dichotomous moderator variable was also created in order to group the respondents according to their strongest sexual-self-meaning instance.

First, we found that both sexual self-instances were related to measures of sexual identity, indicating a possible mediation mechanism that accounts for the effect on identity. The studies investigating Internet sexuality have found that some people feel that they can experience their true sexual selves in more satisfying ways in online interactions, a process that gradually brings about disinhibition and identity transformation with real-life positive consequences, such as more self-acceptance and sexual confidence [10,40]. In the SCD framework, this would mean more coherence between one’s sense of sexual self and one’s sexual needs, values, behaviors and expressions. Consistent with this assumption, the results in this study found that higher levels of SCD decreased the association between the sense of sexual self, components of sexual identity and sexual satisfaction. In other words, not being able to integrate online sexual experiences in a coherent manner was indicative of less relatedness between the sexual self-concept, sexual identity and satisfaction. In this respect, both SCD parameters were significant and produced similar effects. However, significant results were attributed to different outcomes, indicating the possibility that the SCD dimensions could function independently. Additionally, subsequent analyses did not reveal definite corresponding effects for the SCD parameters, further suggesting some degree of separation. This hypothesis is also supported by the fact that the two indexes overlapped only to a certain degree, opening up the possibility for the existence of various profiles of SCD. For example, we could hypothesize that strongly favoring one sexual-self instance over another (SCD distance) might actually be linked to a clearer sense of identity in that context, given low levels of sexual-self inconsistency. However, because of the small sample size for this study, a more detailed analysis of possible data clusters was not viable, and therefore future studies could be aimed at determining the existence and validity of such SCD profiles on sufficiently large samples.

Further insights regarding SCD dynamics were obtained by grouping respondents based on how positively they rated their sense of sexual self in one instance compared to the other (online or offline). This procedure allowed for the emergence of relevant effects of SCD, further supporting the notion that self-concept differentiation does not operate unconditionally [23]. Regression analyses showed that the location of sexual-self had a moderating effect on the relationships of SCD with sexual exploration, sexual-orientation uncertainty, sexual satisfaction and solitary OSAs. The moderating effect was such that high SCD was related to enabling effects for individuals who located their sexual selves online, while the reverse was true for those who located their sexual selves offline. It would seem that, for those more comfortable with offline sexuality, a stronger desire for sexual exploration and more sexual satisfaction was linked to low levels of SCD, therefore to more inter-role consistency and to a higher feeling of comfort in both sexual instances. This trend was maintained also for commitment to a sexual identity and identity synthesis, although the results were not statistically significant. However, a more complicated picture emerged for the remaining subjects, where SCD did not produce significant effects, except in predicting sexual-orientation uncertainty. Even so, the results suggest that for individuals who found it easier to express themselves sexually online, higher levels of SCD seemed to suggest more interest in sexual exploration, greater sexual satisfaction and more frequent engagement in solitary OSAs. It could be that such individuals are in the process of establishing a clearer sense of sexual identity, in which case the greater separation between sexual self-instances could enable the acquisitions necessary for identity transformation and sexual-self coherence. This could also explain why higher levels of SCD were indicative of more frequent solitary sexual activities, such as masturbation and the use of Internet pornography. Given that, for this particular subset in our sample, a higher SCD was related to a less positive sense of sexual self, we could hypothesize that anxiety regarding partnered sexual interactions might be a characteristic of this group; this, in turn, leading to a preference for solitary sexual activities and drawing a positive, yet initially isolated, sense of sexual self from fantasized experiences. Overall, the comparative analysis for the two groups produced mostly non-significant results, but even so, a fairly consistent trend could be observed in the data. Subjects who reported a more positive sense of sexual self in real life rather than online were on average more committed to a sexual identity, more satisfied with their sex lives and had a stronger sense of sexual-self compared to the subjects from the opposite group.

The importance of the sexual self-concept in relation to sexuality-related outcomes has been addressed in previous studies linking various sexual-self components, such as sexual self-esteem or sexual assertiveness, to sexual satisfaction [41,42,43] and identity [11,44]. However, online sexuality still gives way to conflicting views about its impact on behavior and identity, with evidence pointing in both positive and negative directions [40]. First, it is important to note that the results obtained in this study support a profile-based approach on this issue, with self-concept differentiation having the potential to offer additional insight into the complex interplay between the online and offline sexual arenas. For the sample used in this study, SCD seemed to point towards a rather detrimental effect, being more indicative of the self-fragmentation hypothesis, as it was linked to struggles in having a clear sense of sexual identity, regardless of where individuals located their most positive version of sexual self (online or offline). However, it could be that SCD is necessary in enabling the process of identity transformation in those individuals who turn to the online environment for self-expression. SCD did not operate in similar ways for all respondents, indicating that a better understanding of sexual-self instances might play a role in further clarifying the impact and role of SCD in the analyzed framework. Second, some evidence was found in support of a two-dimensional model of SCD (inconsistency and distance), although no definitive conclusions could be drawn for the present sample. Nevertheless, the results obtained in this study encourage further investigation and offer support for using a SCD framework when approaching the complexity of modern sexuality.

### Limitations

The present study has a number of limitations that should be noted. First, conclusions can be drawn only in regard to the association between the measured variables given the cross-sectional approach. The results obtained in this study could be useful in determining new research directions for longitudinal designs aimed at investigating possible transformations in SCD and sexual outcomes based on people’s experience with the online environment. Second, using self-reported measures introduces potential bias in the data, especially given the sensitive topic of sexuality. This type of measurement error can originate in accidental or deliberate misreporting and it also might reflect the respondents’ unawareness of certain aspects of themselves or efforts of conformity to perceived social norms [45]. As such, the results obtained in this study should be considered carefully taking into account the necessity for validation datasets that could generate correction models. A third limitation in this study is related to the specific effects introduced by the cultural and technological landscapes of Romania. Both the general attitudes towards sex that exist in a particular society and the accessibility and affordability of the Internet within that space are important factors to consider when researching the interplay between online and offline sexuality. Knowledge on this topic can be advanced by observing how these phenomena manifest in populations from different cultures with different values and access to technology. Finally, the results obtained in this study should also be interpreted with caution due to the small sample size used. In order to draw more definite conclusions regarding SCD in the online–offline context, larger samples would be needed.

## 5. Conclusions

To our knowledge, this is the first study that attempts to explore self-concept differentiation in the framework of online–offline sexuality. In summary, we determined that SCD was indicative of a discontinuity between the overall sense of sexual self and sexual needs, values and behaviors and satisfaction with sex. We also found that people perceive themselves sexually in a different manner in the online environment than they do in their real lives, with some respondents attributing a more positive meaning to their sexual selves in online sexual experiences. A comparison of the respondents in our sample based on this criterion revealed that locating the sexual-self online was not conclusively linked to better sexual outcomes, such as a clear sense of sexual identity or sexual satisfaction. However, this particular case could reflect an initial step in the process of identity transformation and sexual-self normalization. Indeed, the findings indicate that those individuals who felt more positive about themselves as sexual beings in real-life, rather than online, also had a clearer sense of their sexual identity, were more satisfied with their sex life and were more social in their approach to online sexuality. Although some results did not reach statistical significance, the observed trend in the data encourages further investigation. Also, we found some evidence that the degree of self-differentiation had particular associations with aspects of sexual identity and sexual satisfaction that were dependent upon where the more positive sense of sexual self was localized. Additionally, we set out to explore the possibility that the two ways of expressing SCD, as non-shared variance or absolute differences, could in fact reflect two separate dimensions that may vary independently to certain degrees and have specific effects. However, in the present study, we did not find sufficient evidence in this regard and we encourage further investigations of this topic with more adequate approaches, such as cluster analysis. In conclusion, the results in this study offer promising evidence for the SCD model being a useful tool in explaining and understanding various phenomena associated with how people view and express themselves sexually in today’s complex reality.

## Figures and Tables

**Table 1:** Correlation matrix and descriptive statistics.

	1	2	3	4	5	6	7	8	9	10	11	12
1. SCDI	—											
2. SCDD	0.593 ***	—										
3. Online SS	−0.373 ***	−0.677 ***	—									
4. Offline SS	−0.133	−0.186	0.577 ***	—								
5. SOSAs	−0.037	−0.043	−0.074	−0.197 *	—							
6. POSAs	−0.239 *	−0.114	0.066	0.092	0.389 ***	—						
7. ESSx	−0.007	0.04	0.422 ***	0.613 ***	0.032	0.103	—					
8. PSSx	−0.153	−0.101	0.428 ***	0.498 ***	−0.066	0.051	0.769 ***	—				
9. Exploration	−0.169	−0.186	0.380 ***	0.230 *	0.202 *	0.232 *	0.393 ***	0.410 ***	—			
10. Commitment	−0.112	−0.161	0.459 ***	0.698 ***	−0.102	0.14	0.624 ***	0.495 ***	0.208 *	—		
11. Synthesis	−0.052	−0.056	0.434 ***	0.551 ***	−0.058	0.178	0.523 ***	0.418 ***	0.355 ***	0.695 ***	—	
12. Orientation	0.016	0.069	−0.322 ***	−0.548 ***	0.235 *	0.051	−0.374 ***	−0.269 ***	0.117	−0.66 ***	−0.453 ***	—
Mean	0.608	0.625	91.028	97.292	6.792	3.057	24.915	22.934	35.094	29.198	24.198	5.642
SD	0.312	0.637	20.924	17.511	3.029	3.569	4.737	5.949	9.254	5.848	4.701	3.594

Note. SCDI = SCD Inconsistency; SCDD = SCD Distance; SOSAs = Solitary OSAs; POSAs = Partnered OSAs; ESSx = Ego-focused sexual satisfaction; PSSx = Partner-focused sexual satisfaction; SD = Standard deviation; * *p* < 0.05, *** *p* < 0.001.

**Table 2:** Group comparison—ANOVA—means and standard deviations.

	LSS Offline	LSS Online	F
	n = 63	n = 32	
Solitary OSAs	6.71 (3.07)	7.00 (3.15)	0.180
Partnered OSAs	3.30 (3.67)	2.46 (3.17)	1.193
Commitment to a sexual identity	29.76 (5.69)	27.28 (6.11)	3.829 *
Interest in sexual exploration	34.41 (9.59)	35.43 (9.39)	0.246
Sexual-orientation uncertainty	5.39 (3.62)	6.46 (3.79)	1.801
Sexual-identity synthesis	24.00 (4.81)	23.75 (4.48)	0.060
Ego-focused sexual satisfaction	25.50 (4.18)	23.50 (5.69)	3.808 *
Partner-focused sexual satisfaction	23.38 (5.81)	21.81 (6.50)	1.426
Online sexual self	87.38 (22.56)	93.62 (17.11)	1.893
Offline sexual self	101.84 (14.71)	85.90 (18.36)	20.983 ***
SCD inconsistency	0.65 (0.28)	0.61 (0.32)	0.266
SCD distance	0.75 (0.71)	0.56 (0.45)	1.964

Note. * *p* < 0.05; *** *p* < 0.001.

## Data Availability

Results and dataset can be made available by request.
